# General Principles to Justify Plant Biostimulant Claims

**DOI:** 10.3389/fpls.2019.00494

**Published:** 2019-04-16

**Authors:** Manuele Ricci, Lorraine Tilbury, Bruno Daridon, Kristen Sukalac

**Affiliations:** ^1^Independent Researcher, Perugia, Italy; ^2^European Biostimulants Industry Council, Prospero & Partners, Antwerp, Belgium; ^3^Olmix Group, Bréhan, France

**Keywords:** plant nutrition, plant biostimulant, agronomic claim, nutrient efficiency, abiotic stress, crop quality, yield, trial design

## Abstract

The forthcoming European Union (EU) Fertilizing Products Regulation^1^ proposes a claim-based definition of plant biostimulants, stipulating that “plant biostimulant” means a product stimulating plant nutrition processes independently of the product’s nutrient content, with the aim of improving one or more of the following characteristics of the plant: nutrient use efficiency, tolerance to abiotic stress, crop quality traits or availability of confined nutrients in the soil and rhizosphere. The future regulation also specifies that a plant biostimulant “shall have the effects that are claimed on the label for the plants specified thereon.” This creates an onus for manufacturers to demonstrate to regulators and customers that product claims are justified. Consequently, the justification of the agronomic claim of a given plant biostimulant will be an important element to allow it to be placed on the EU market once this new European regulation is applied. In this article, members of the European Biostimulant Industry Council (EBIC) propose some general guiding principles to follow when justifying plant biostimulant claims, that are outlined in this article. These principles are expected to be incorporated into harmonized European standards that are being developed by the European Committee for Standardization (CEN) to support the implementation of the regulation.

## Introduction

The forthcoming EU regulation for fertilizing products covers six types of products (fertilizers, liming materials, soil improvers, growing media, inhibitors, and plant biostimulants) as well as combinations of them. The definition used for plant biostimulants is claims-based (European Commission, 2016; Council of the European Union, 2018), meaning that it is the function of the product, not what it contains that defines it as a plant biostimulant. For this reason, demonstrating that a product is indeed a *bona fide* biostimulant depends on a demonstration of its effect. However, this should not be confused with guaranteeing a specific level of efficacy. In no case should the placing of a biostimulant on the EU market be considered to guarantee effectiveness under all conditions, as many factors may influence performance of a biostimulant in the field.

The requirements for claims justification should be proportional to the task; manufacturers should provide enough data to be credible, without the process becoming needlessly burdensome. Sound scientific support of product claims is one of the central tenets of the European Biostimulant Industry Council’s (EBIC) work, and EBIC has taken inspiration from how other sectors justify product claims. However, existing claim justification protocols for other product categories cannot be automatically applied to biostimulants in a copy/paste manner, for several reasons:

• the diversity of existing biostimulant products (including microbial and non-microbial products) and the subsequent wide range of possible claims;
• the dependence of biostimulant effects on multiple contextual factors, and
• the interactions among biostimulant components as well as between the biostimulant itself and other systemic elements (e.g., weather, soil microbiome, soil type, crop variety, etc.).

In order to promote high-quality data supporting biostimulant claims – whether for placing on the market or for commercial purposes – EBIC engages with key stakeholders in the co-development of guidelines for compiling robust data on biostimulants that favor consistent and reliable results.

European Biostimulant Industry Council has identified a need for guidelines to address two levels of producing scientific argumentation for claims:

(1) Generating data to support a biostimulant claim;
(2) Using data either to support placing a biostimulant on the EU market and/or to support commercial product claims.

How much data is required to support a claim? It depends on the claim. The narrower the claim, the less data should be required. Given the variety of possible effects, crops or crop groupings, and growing conditions, manufacturers need the flexibility to design studies that are adapted to the specific agronomic situation. Furthermore, it should be recognized that when it comes to products that improve the availability of nutrients (notably micro-organisms), soil types and conditions may be more relevant than crop types themselves. Design flexibility is therefore needed to accommodate such cases.

## Ebic’s Functional Definition of Biostimulants

European Biostimulant Industry Council uses a functional definition to describe biostimulants that was developed over the course of a year-long consultation process with stakeholders including researchers, regulators and related industry sectors.

“Plant biostimulant means a material which contains substance(s) and/or microorganisms whose function when applied to plants or the rhizosphere is to stimulate natural processes to benefit nutrient uptake, nutrient efficiency, tolerance to abiotic stress, and/or crop quality, independently of its nutrient content.”

The definition was crafted to respond to several needs as outlined below:

... a material which contains substance(s) and/or microorganisms (including microalgae) (Chiaiese et al., 2018) – Because there are system effects when substances and/or micro-organisms are combined, biostimulants can only be accurately defined and evaluated at the level of the final formulation (notwithstanding incorporation into another product like a fertilizer or a growing medium). Internal surveying of EBIC’s members reveals a trend toward complex multi-component products rather than products with only one (or one type) of biostimulant substance (European Biostimulants Industry Council [EBIC], unpublished). Indeed, it is precisely the synergistic effects among different types of biostimulants (microbial/non-microbial, substances of different origins, etc.) allow manufacturers to design and develop efficient plant biostimulant products with specific properties in terms of yield and especially nutritional and functional quality (Krouk, 2015; Rouphael and Colla, 2018).
... whose function – EBIC advocates for a functional use definition because this corresponds best to how products are placed on the market and the form in which farmers use them. This is in contrast with a *substance-based definition* that is based on the chemical or biological identity of the components (or of a single component considered in isolation from the others) or with a *mode-of-action definition* that is based on the mechanism through which the effect is obtained. The problem with definitions based on content or mode of action is that more than one effect may be related to an ingredient or a mode of action, and those effects may fall under different regulatory frameworks. *The functional use definition is intimately tied to the claim and use of the product, both of which can be verified through post-marketing surveillance*. Therefore, a functional use definition is the most practical because it provides the information needed by final users and provides a basis for controlling appropriate marketing and sales.
... when applied to plants or the rhizosphere –Foliar applications are used for some biostimulants, but others are applied directly to the soil (or other growing medium) or as a seed treatment.
... to stimulate natural processes – Many of the processes that biostimulants influence are inside the plant, but others occur in the soil (or other growing medium) around the plant. Soil processes may be particularly relevant for microbial products, lixiviates, humic acids, organic matter derivates, etc. which are often applied to soils rather than for other biostimulants that are applied through foliar application.
... to benefit nutrient uptake, nutrient efficiency – Nutrient uptake is related to, but distinct from, nutrient efficiency. The latter covers how well the plant uses a nutrient once it has been made available. The former can include the effects of biostimulants that make nutrients more available, for example through biological fixation of nitrogen or solubilization of phosphorus.
... tolerance to abiotic stress – Because biostimulants affect plants’ general well-being, it makes them more resilient to harsh growing conditions. Specific products may also provide the plant with specific tolerance against an abiotic stress, such as drought, salinity or extreme temperatures.
... and/or crop quality – Like all products related to plant nutrition and soil fertility, biostimulants can directly influence crop quality. They do this in many ways, for example, by increasing the plant’s access to essential nutrients or reducing the energy used by the crop in times of stress. These effects generally also have a positive influence on yield.
... independently of its nutrient content – Biostimulants may contain mineral elements found in fertilizers but are applied at such low doses that the benefit cannot be attributed to a fertilizer effect, and indeed trials can demonstrate that the nutrients present are not the cause of the observed effect.

Although not part of the product definition, the functions provided by biostimulants have a role to play in “agro-ecology,” i.e., the application of ecological principles to agriculture and the food production system (Table 1; IFOAM EU Group et al., 2012).

**Table 1:** Agro-ecological principles and the role played by biostimulants.

Increase biodiversity
By improving soil micro-organism quality/quantity
**Reinforce biological regulation and interactions**
By reinforcing plant–micro–organism interactions
– symbiotic exchanges i.e., *mycorrhizae*
– symbiotic exchanges i.e., *rhizobiaceae/fava*
– secretions mimicking plant hormones (i.e., *trichoderma*)
By regulating plant physiological processes
– e.g., growth, metabolism, or plant development
**Improve biogeochemical cycles**
– improve absorption of nutritional elements
– improve bioavailability of nutritional elements in the soil
– stimulate degradation of organic matter

## Claim Justification Should Focus on the EU Definition of Biostimulants

With a view to placing products on the European market, all claims should be demonstrated regarding the agreed EU definition of a biostimulant, per one of the four categories indicated in the forthcoming EU Fertilizing Products Regulation (European Commission, 2016; Council of the European Union, 2018): improving nutrient use efficiency, tolerance to abiotic stress, crop quality traits or availability of confined nutrients in the soil and rhizosphere.

Because each of these categories of claims is quite broad, a more precise description of claims as illustrated in Table 2 allows for specific measurements to be defined and conducted.

**Table 2:** Categories of biostimulant claims and examples of sub-claims.

Category of claims in the forthcoming fertilizing products regulation	Examples of sub-classes
Nutrition efficiency improvement	Phosphorus acquisition improvement, yield increase
Abiotic stress tolerance	Salt stress, drought stress
Crop quality improvement	Potatoes size increase, sugar content increase, storage duration improvement as the result of improved quality (e.g., firmness, for ex), processing improvement
**Derived claims**	
Yield improvements	Yield increase or yield security

Furthermore, effects are often translated into claims that have value for farmers, such as increased yield (which is not specifically mentioned in the forthcoming EU Fertilizing Products Regulation but could be the result of the use of any of the products it covers) and improved crop quality (which is specifically mentioned as an effect of biostimulants). Although yield gains can be traced back to one of the underlying biostimulant claims defined in the regulation, EBIC has listed them as a separate category of claim, to ensure that they are measured and reported in appropriate empirical terms.

## Type of Information That Can Support a Claim

Various types of data and empirical evidence can support claim justification. While not strictly speaking hierarchical, it makes sense to begin with published literature and existing data and then to complement that information as needed with new experimental data from controlled conditions and field trials (Rouphael et al., 2018).

Data generated under controlled conditions (glasshouse, growth chambers, phenotyping, etc.) from outside the European Union should be admissible if the climatic conditions tested could conceivably apply within the EU and:

• if it is from a manufacturer’s own GEP/GLP-certified facility;
• if the independent research partner (contract facility, university, etc.) that generated the data is considered reputable, or
• if the manufacturer can otherwise demonstrate that the quality of the methodology and the data obtained are substantially equivalent to what would be achieved by a GEP/GLP facility.

### Use of Published Literature and Existing Data

As mentioned in the beginning of the Section “Type of Information That Can Support a Claim” of this publication, peer-reviewed scientific literature can support a claim. Literature can be used to describe the mode of action of the product, the biology of the microorganisms used, or any preliminary studies described in relevant published papers supporting the basis of the proposed claim. Scientific literature can be used to support a claim if it is of acceptable quality, for example, evaluated per criteria outlined in Klimisch et al. (1997) and used to determine which literature can be acceptable for data requirements under EU chemical legislation (REACH). At the same time, the synergistic or emergent effects that result from the combination of substances within a product (Krouk, 2015; Rouphael and Colla, 2018) mean that it is unlikely that literature alone will be enough to fully justify a claim.

Existing scientific information and existing field trial data should be evaluated per the criteria for relevance, reliability, and adequacy defined by the European Chemicals Agency (ECHA) for the implementation of the EU’s chemicals regulation (REACH) (European Chemicals Agency [ECHA], 2011).

### Experimental Data

Biostimulant claims can be supported by experimental data generated under controlled conditions (laboratory, greenhouse, growth chamber, phenotyping, etc.) and/or in the field (field trials). Additional data from carefully designed small-scale laboratory and growth chamber studies will often form a vital component of the overall claim justification package provided. If field data are used, at least some EU data must be included. Field data from outside the EU may support EU data if both are generated under similar geo-climatic conditions (and those correspond to the intended context for product use). Guidelines exist for determining the comparability of geo-climatic conditions (European and Mediterranean Plant Protection Organization [EPPO], 2014).

Field trials provide essential information about biostimulant effects under real-world conditions. However, for some claims, such as salt stress or cold stress, it is difficult to artificially create the appropriate field conditions. In such cases, the focus of field trials would be more on the “holistic” benefits of the biostimulant in terms of yield/quality, while a specific biostimulant claim related to its mode of action could be demonstrated in controlled conditions. For example: one could demonstrate that phosphorous solubilization occurs in controlled greenhouse or laboratory conditions when the biostimulant product is used, and, demonstrate the overall general benefit to the farmer in terms of improved yield and/or quality when field testing the same product. The field trial would not be needed if the manufacturer only wanted to claim the phosphorus solubilization without mentioning the subsequent improved yield and/or quality. (Although the farmer doesn’t care about the mode of action if it doesn’t result in a tangible on-farm benefit.)

The net agricultural benefit after considering both the positive and negative effects of the biostimulant should be large enough to justify its use. The benefit from the use of a product should be appropriate to the agronomic setting in which the product will be used. A low level of benefit may be acceptable in some situations, for example:

• when a product will be used as a component of an Integrated Crop Management (ICM) program, or
• in specialized situations, such as organic farming, or where the product makes it possible to maintain the same level of yield or quality while decreasing nutrient applications.

Where the data indicate that there are significant inconsistencies in the performance of a product, the reasons for these inconsistencies should be explained. The instructions for use should enable the user to identify the conditions under which the product will provide optimal performance and any factors that may have an impact on effectiveness. Unexplained variations in product performance should not be a barrier to placing the product on the market, but the uncertainties of the product claim should be indicated on the product label in that case. Transparency is critical to allow farmers to make informed choices.

#### General Guidelines for Trials/Assays of Biostimulants

Use of a statistical program should be adapted to field trial software such as ARM.

##### Trial study plan

The trial study plan should cover the following topics:

• The aim of the trial series;
• Statistical analysis and trial design;
• Trial conditions;
• Design and lay-out of trials;
• Control data;
• Application of treatments, and
• Mode of assessment.

###### The aim of the trial series

• Objective of the trial and basic information on the trial site. The objective of the trial should be specified, including:
  ˚ The biostimulant effect(s) to be demonstrated
  ˚ The inclusion of other variables in the trial (dose rates, application conditions)
• Whether the trial is for evaluation of a claim or another purpose (germination test, quality of the harvested product, effects on succeeding crops, etc.).
• The following basic information should be provided on the trial site:
  ˚ Full address and geographical coordinates, if possible;
  ˚ Crop and cultivar;
  ˚ Any useful details on the site (e.g., exposure and slope).

###### Statistical analysis and trial design

The placing of a biostimulant on the market should never be considered to guarantee effectiveness under all conditions, as many factors may influence the performance of a biostimulant in the field. Many additional factors are relevant to biostimulant products when determining acceptable, beneficial, levels of action. These can include:

• Offering an approach compatible with ICM systems and/or organic farming;
• Greater compatibility with cultivation practices;
• Mitigating undesirable effects (on human beings, beneficial organisms, non-target organisms, other crops etc.) of the alternative production system;

In such cases, biostimulant manufacturers should ensure that users can be provided with accurate information on the likely performance of the product and advice on how best to use the product so that it will perform as effectively and consistently as possible.

Under controlled conditions (greenhouse or laboratory), the level of confidence compared to an untreated control can be set at 90% probability (minimum for agronomic production trials in controlled conditions), given the nature of biostimulant effects and their inherent variability (physiology and biology sensitive to pedo-climatic conditions, local microbiome biodiversity and crop genetics).

A minimum of three field trials in the EU should be performed to demonstrate the desired biostimulant claim. The observation of consistent “agronomically” positive data trends (i.e., not necessarily statistically significant) compared to untreated plots in field trials could be considered sufficient to justify a biostimulant claim.

Trials do not need to be over multiple seasons if there are enough trials in one season and different geo-climatic conditions pertinent to the environmental conditions and relevant to the agronomic conditions for which the product will be sold to justify the claim. The trials should be conducted in the EU.

Trial design should be done in a way that allows to discriminate between treated and untreated plots It is recommended to have enough replicates to ensure to reduce the variability of the data and increase the chance to see consistently differences with the untreated plots.

Where a biostimulant’s performance is affected by temperature, soil type, crop, or other parameters, the trial design, execution, and subsequent user recommendations should take these factors into account. Furthermore, some claims may be better tested under controlled conditions (e.g., abiotic stresses may be difficult to induce in the field).

When designed accordingly, multiple claims may be demonstrated in a single trial.

Untreated plot trial results in terms of yield and quality should reflect normal agronomic expectations for local production.

The study plan should specify what is assessed, how it is assessed, when, and why.

It is recommended to evaluate at least one measurable indicator related to a benefit perceived by the farmer (i.e., impact on yield and/or quality). For example, nodulation is an early measurable parameter, but it is not sufficient on its own to confirm an effect on yield and/or quality of the crop. If an indicator of biostimulation is not assessed in the trials designed to justify a claim, then this indicator cannot be claimed on the label. For example, a claim to increase root growth is measured and demonstrated in an experimental trial, but the crop yield increase is not quantified in the trial. Consequently, the label can claim “increased root growth” but cannot claim “improved yield” because an increase in yield was not quantified in the claims justification data.

Phytotoxicity should be verified during trials. If no evidence of phytotoxicity is observed, then there is no need to conduct additional phytotoxicity trials. However, if signs of phytotoxicity are observed, then phytotoxicity data are needed. At a minimum, the manufacturer should provide recommendations on how to minimize phytotoxicity when the biostimulant is used.

Trials must be conducted by qualified personnel who will record, document, and archive the trial study plan, the results, the final report and all the supporting raw data. The Fertilizing Products Regulation specifies that manufacturers must keep such information at the disposal of national authorities for 5 years after the product has been placed on the market to facilitate market surveillance. Good Experimental Practice (GEP) for plant protection products call for such information to be archived for 10 years (European and Mediterranean Plant Protection Organization [EPPO], 2012); however, this may be influenced by the record-keeping requirements found in the EU’s plant protection regulation (European Parliament and of the Council, 2009).

Data from GEP/GLP-certified facilities can be considered credible, even if they belong to the manufacturer. Nonetheless, it is desirable that manufacturers can demonstrate that at least some of the research was conducted with impartial and competent third parties. As much as possible, trials to support product claims should be conducted with an independent and competent partner, such as one of the following:

• National research agencies and extension officers;
• Institutes (including but not limited to universities and other institutes of higher learning and private research stations) and researchers with published research in agriculture and agronomy, and
• Certified private research centers (GLP/GEP or GLP/GEP-equivalent conditions).

###### Trial conditions

The relevant conditions of the plot and crop should be adequately described, for example:

• For an annual crop, sowing or planting date and density, row spacing;
• For a perennial crop, arrangement and spacing in rows or as single plants, pruning or training system, rootstock, canopy height, plant width, age, whether in production;
• For a glasshouse crop, arrangement within compartments, on benches, in soil-less culture, etc.;
• The cultivation practices of the crop could be described, such as tillage, fertilizer and irrigation regimes, and any other additional inputs;
• Information should be given on whether the crop was growing normally or was under stress at the time(s) of treatment [e.g., drought, frost, wind or effects of other overall chemical treatments, and/or effects of other pests (including diseases and weeds)];
• For a soil-applied product, the temperatures at the root zone level in topsoil should be recorded during at least the first month of the trial at 2-h intervals), and
• Soil characteristics should be described, i.e. the percentage of sand, clay, silt, and Organic Matter (O.M.), as well as the pH.

###### Design and lay-out of trials

The design and lay-out of the plots should be described, preferably with a plan, the number, size and shape of plots, whether defined by plot dimensions on the ground or a certain lay-out of plants.

The type of experimental design should be indicated. The arrangements made for the untreated control (included, imbricated, and excluded) should be precisely indicated, together with details on any other control treatments.

Completely randomized blocks should be assured, while maintaining a scientific design that avoids any interference of experimental conditions between plots (for example, with regards to drought stress mitigation trials, the well-watered condition will have to be set up as a band reference beside the trial.

Enough replicates should be assured to obtain 12 degrees of freedom in the trial, so that a consistent difference between treated and untreated crops can be demonstrated.

###### Control data

Control data set can be completely untreated plot or an “omission” group i.e., the treatment regimen is the same with the exception of the biostimulant, which is absent from the “omission” plot.

Control object(s) selected for an improved nutrient uptake claim should include:

• Untreated
• The following additional control groups, when a biostimulant is included in a “support” nutrient-containing formulation:
  ˚ option 1: the support formulation alone, if the support provides nutrient elements;
  ˚ option 2: the biostimulant formulation alone (without the nutrient elements).
• Abiotic stress resistance claims should include the following control objects:
  ˚ Stress condition object(s);
  ˚ no-stress condition object(s);
  ˚ characterization of the applied stress level.

###### Application of treatments

Precise information should be provided on the formulation, application method, concentration and amounts of the test product.

The justification of biostimulant claims should be done for the minimum recommended dose necessary to achieve the desired effect. However, for biostimulant products composed primarily of substances that occur naturally in the environment, this may be less important, unless the additional amount significantly increases existing background levels. Additionally, for some biostimulants (microbial biostimulants for example), the concept of a minimum effective dose may be more difficult to determine practically, and a range of doses may be more appropriate to justify the associated claim. In such cases, while an appropriate explanation for the proposed dose is required, providing field-generated data may not be necessary. Such explanations should refer to the mode of action, and to the biology of the microorganisms. One may also include any preliminary studies (including relevant published peer-reviewed papers) that provide the basis for the proposed concentration in the formulation and/or applied dose.

While manufacturers should always seek to justify the recommended dose, the lack of precise or conventional dose justification data should not preclude the placing of the biostimulant on the market. However, in such cases, the manufacturer should explain why such a dose may not be appropriate. Information demonstrating the minimum level required to provide a beneficial effect (as determined for effectiveness, in either laboratory or field studies) may suffice.

• Test and reference products: the products included in the trial (test and reference) should be specified, giving the common name or other specified standard (if available), and the exact name or other designation of each formulated product.
• Mode of application: The information provided should be sufficient to establish that good agricultural practice is being followed, for example:
  – The application method and equipment used;
  – Any significant deviations from the intended dosage;
  – The operating conditions, insofar as they may affect claims (e.g., for sprays, pressure, nozzle type, spray quality and speed of travel of sprayer);
  – The number of applications made;
  – The date of each application (including year, preferably by dd-mm-yyyy);
  – The growth stage of the crop at the time of each application (see BBCH Growth Stage Keys Meier et al., 2009);
  – The doses used (cc-g/hL or L-Kg/ha), and the spray volumes (L/ha).
• Meteorological data. The following meteorological data should be recorded:
  – Observations by the experimenter near the date of application of meteorological data that may affect the outcome of the trial. These depend on the judgment of the experimenter and need not be given at the same level of detail as on the day of application.
  – Observations made by the experimenter on the day of application, including certain standard data that should always be provided for the application day (temperature, humidity and wind); if rain occurred within 24 h of the foliar application of the product, it should be recorded as “yes/no” (rain fastness)
• Edaphic data should also be recorded during the trial.

###### Mode of assessment

• Type, time and frequency of assessment
• The type and date of each assessment
• The methods used should be described. Any assessment scales used should be specified.
• Direct effects on the crop. The presence or absence of phytotoxic effects should be noted for each plot, with an accurate description of any symptoms, for example: modifications in the development cycle, thinning, modifications in color, necrosis, deformations, effects on the quantity and quality of the yield.
• Yield and quality should, when specified, be recorded, taking careful note of the specific parameters required in each crop.

##### The trial series report

The trial report should include:

• The aim of the trial series;
• The list of test and reference products, with doses and application times of frequencies;
• The assessment methods;
• Results including statistical analysis if any were conducted;

##### Crop groupings for the conduct of biostimulant field trials

When trials are conducted to justify biostimulant claims, the crop groupings outlined in Table 3 are proposed.

**Table 3:** Proposed crop groupings to justify biostimulant claims.

• Cereals (wheat, barley, oat, rice, minor grains) and corn
• Pulses and oilseeds
• Tree fruit, nuts, and olive
• Grape (wine and table)
• Other soft fruit and vegetables (all leafy, fruiting and root vegetables, and leguminosae)
• All others [loam (turf), ornamentals, and mushrooms, etc.]

European Biostimulant Industry Council suggests the guideline proposed in Table 4 to help manufacturers determine the appropriate number of trials, depending on the nature of the claim and to prevent excessive requests for trials from reviewing authorities. Notwithstanding this guideline, manufacturers will need to adapt their trial regime to the specific claim being made, especially as several of the examples listed in Table 4 may apply.

**Table 4:** Suggested number of trials based on the claim to be justified.

Claim that can credibly be made on this basis	Suggested number of trials
Effect claimed for *a specific crop* Example: Improves strawberry ripening	3 trials on the crop Example: Product is successfully demonstrated on strawberries in the field in a single location over 2 years or tested in the field and in a commercially equivalent growing environment the same year.
Effect can be claimed for *the entire crop group* Example: improves brix content or yield of larger grade fruit	3 trials each for 2 different crops or 2 trials for 3 each diff crops within a single group. Example: Product is successfully demonstrated on apples and pears in a single location over 2 years or in two different locations with different growing conditions in a single year.
Effect can be claimed *without being required to limit it to any specific crop grouping* Example: Helps crops tolerate drought stress in open-air commercially equivalent growing environments	3 trials each from 4 different groups. Example: Product is successfully demonstrated on cereals, apples, grapes, and peppers.

Where applicable, appropriate scientific literature may be substituted for one or more of the trials suggested.

## Conclusion

The ability to demonstrate that a product is indeed a *bona fide* biostimulant will depend on a demonstration of its effect. However, this should not be confused with guaranteeing a specific level of efficacy. In no case should the placing of a biostimulant on the EU market be considered to guarantee effectiveness under all conditions, as many factors may influence performance of a biostimulant in the field. The requirements for claims justification should be proportional to the task; manufacturers should provide enough data to be credible, without the process becoming needlessly burdensome. The narrower the claim, the less data should be required. Given the variety of possible effects, crops or crop groupings, and growing conditions, manufacturers need the flexibility to design studies that are adapted to the specific agronomic situation. Furthermore, it should be recognized that, in the case of products that improve the availability of nutrients (notably micro-organisms), soil types and soil conditions can be more relevant than crop type itself when designing trials. Trials will become ever more crucial as the industry trends toward the development of complex, multi-component products. Demonstrating multiple effects, especially when they are synergistic or emergent, will provide additional challenges for developing appropriate trial designs.

The forthcoming EU regulation is based on the New Legislative Framework, which means that harmonized standards play an important role during the conformity assessment process; measures obtained through the application of these standards are presumed to be in conformity with essential requirements of products both for safety and quality, if the values are within any applicable target ranges. Such harmonized standards play a role in characterization, verifying contaminant levels, declared contents and product claims.

The European Committee for Standardization (CEN) has begun work on processes and methods that will become the basis of a set of harmonized European standards to justify biostimulant claims (CEN and CENELEC Work Programme, 2018) under the forthcoming EU Fertilizing Products Regulation. This work includes the standardization of denominations, biostimulant specifications, marking, test methods, verifying product claims and safety requirements. It will be a challenge to develop standards that allow for comparability of products (i.e., knowing that two different products truly address the same abiotic stress, for example) while accommodating the wide range of products, claims and contexts for use.

The guidelines developed by EBIC and outlined in this article can inform the drafting of relevant CEN standards on claims justification, as they have already benefitted from significant discussion among professionals involved in the testing of biostimulant product claims.

## Author Contributions

All authors listed have made a substantial, direct and intellectual contribution to the work, and approved it for publication.

## Conflict of Interest Statement

At the time this article was drafted and submitted, MR was employed by Bayer CropScience S.r.l., a member of the European Biostimulant Industry Council (EBIC). BD was employed by Olmix Group, another EBIC member. KS is a Consulting Partner at Prospero & Partners, which is contracted to provide the EBIC secretariat. LT is an independent consultant working for Prospero under contract to support EBIC on technical issues.
